# Effects of Noisy Galvanic Vestibular Stimulation During a Bimanual Tracking Robotic Task

**DOI:** 10.3389/fnins.2019.01132

**Published:** 2019-10-25

**Authors:** Bulmaro A. Valdés, Carlo Menon

**Affiliations:** Menrva Research Group, Schools of Mechatronic Systems and Engineering Science, Simon Fraser University, Metro Vancouver, BC, Canada

**Keywords:** noisy galvanic vestibular stimulation, robotics, stochastic resonance, upper extremity, bimanual

## Abstract

**Background:**

Noisy galvanic vestibular stimulation (nGVS) has been shown to improve motor performance in people with and without disabilities. Previous investigations on the use of nGVS to improve upper-limb motor performance have focused on unimanual fine motor movements, nevertheless, bimanual gross movements are also essential for conducting activities of daily living and can be affected as a result of cerebral dysfunction. Consequently, in this study we investigated the effects of nGVS on bimanual gross motor performance.

**Methods:**

Twelve healthy participants completed a visuomotor task in which they performed bimanual upper-limb movements using two robots. During the task, participants tracked a target that oscillated following a sinusoidal amplitude-modulated trajectory. In half of the trials, participants received subthreshold nGVS, in the other half, they received sham stimulation. Primary outcome measure: percent improvement in root mean square error (RMSE) between the target’s and cursors’ trajectories. Secondary outcome measures: percent improvement in lag between the cursors and target; and percent improvement in RMSE between the cursors’ trajectories. A post-test questionnaire was administered to evaluate the experience of participants.

**Results:**

Tracking error was not affected by nGVS: left −2.6(5.5)%, *p* = 0.128; right −0.9(6.2)%, *p* = 0.639; nor was bimanual coordination −1.5(9.6)%, *p* = 0.590. When comparing if one hand was affected more than the other, we did not find a statistically significant difference (−1.7(3.3)%, *p* = 0.098). Similar results were found for the lag. Questionnaire results indicated that the robotic devices did not limit participants’ movements, did not make participants feel unsafe, nor were they difficult to control. Furthermore, participants did not feel unsafe with the nGVS device, nor did they report any discomfort due to nGVS.

**Conclusion:**

Results suggest that nGVS applied to people without disabilities do not affect bimanual gross motor performance. However, as this was the first study to investigate such effects, stimulation parameters were based on previous unimanual fine motor studies. Future studies should investigate optimal stimulation parameters for improving upper-limb gross motor performance. Overall, participants felt safe using the robotic devices and receiving the noisy electrical stimulation. As such, a similar setup could potentially be employed for subsequent studies investigating the relation between upper-limb performance and nGVS.

## Introduction

Noisy galvanic vestibular stimulation (nGVS) is a non-invasive electrical stimulation technique in which a noise signal is delivered through the mastoids (Pan et al., 2008). nGVS has been shown to have beneficial motor effects in different activities, such as standing balance and walking performance in people with and without bilateral peripheral vestibular dysfunction (Iwasaki et al., 2014, 2018; Wuehr et al., 2016; Inukai et al., 2018) and upper-limb fine motor performance in people with and without Parkinson’s disease (Kim, 2013; Lee et al., 2015; Kuatsjah et al., 2019). In terms of non-motor effects, nGVS has been shown to speed visual memory recall in people without disabilities (Wilkinson et al., 2008). It has been proposed that some of the potential beneficial effects of nGVS are in part due to the mechanism of stochastic resonance (Lee et al., 2015; Fujimoto et al., 2016; Wuehr et al., 2016). The concept of stochastic resonance suggests that by adding noise to a non-linear system, the information content of a signal or the detection threshold of stimuli can be enhanced (Moss et al., 2004). The aforementioned studies, together with the relative safety, low-cost and portability of the stimulation systems, make nGVS an attractive technique to augment or restore function.

Upper-limb movements can be affected by cerebral dysfunction as a result of conditions such as stroke (Kwakkel et al., 2003) and Parkinson’s disease (Flash et al., 1992). Thus, the investigation of effects of nGVS on upper limb motor performance is of particular interest to assess its potential as a clinical tool. Previous studies on upper-limb performance employed visuomotor tasks, as a means to gain insight into human neuromotor response (Ryu and Buchanan, 2012), and focused on the effects of nGVS on unimanual fine motor movements of people with and without Parkinson’s disease (Kim, 2013; Lee et al., 2015; Kuatsjah et al., 2019) reporting positive effects on motor performance. In contrast, it has also been reported that sub-threshold non-noisy GVS deteriorates left arm horizontal position sense in right-handers (Schmidt et al., 2013a, b), and that subthreshold and suprathreshold pseudo-random sinusoidal GVS does not affect unimanual tracking performance (Dilda et al., 2012). Despite the importance of fine motor skills, several basic activities of daily living (Mlinac and Feng, 2016) (e.g., eating, dressing, and bathing) also require gross upper-limb function and the use of not one but both limbs (Lum et al., 1993). Moreover, given that older adults [high risk of developing Parkinson’s disease (Reeve et al., 2014) and/or having a stroke (Kelly-Hayes, 2010)] tend to perform tasks with both hands (Kilbreath and Heard, 2005), the inclusion of bimanual exercises, in addition to unimanual, might be of importance for rehabilitation interventions (Wolf et al., 2014). Consequently, we proposed to investigate the effects of nGVS on upper-limb bimanual gross motor performance to gain insight into the impact of this technology on larger arm movements that involve both limbs.

In this study, we employed a robotic platform to objectively capture participants’ gross motor movements, while allowing them to interact with the computer’s visuomotor task. Furthermore, robotic devices have been proposed as potential upper-limb rehabilitation tools for people with disabilities (Maciejasz et al., 2014; Valdés and Van der Loos, 2018). As such, their combination with galvanic vestibular stimulation (GVS) could allow for new therapy paradigms, akin to the ones that have been proposed for combined transcranial direct current stimulation and robotic devices (Edwards et al., 2009; Straudi et al., 2016). To our knowledge, this is the first study to investigate the effects of nGVS on a robotic bimanual upper-limb gross motor task.

The research questions for this behavioral study were: does nGVS affect spatial or temporal upper-limb gross motor performance, and is one hand more affected than the other while participants receive nGVS? In this work we focused on investigating the impact of nGVS in people without disabilities as an initial step to gain a better understanding of how this technique affects gross motor upper-limb performance before it can be tested in clinical populations.

## Materials and Methods

### Participants

Twelve healthy right-handed adults (six females and six males; Age 32(9) years old) participated in the study (research laboratory setting) after providing informed written consent. The study was approved by the university’s Research Ethics Board. Exclusion criteria included: allergy to rubbing alcohol and/or conductive paste/gel; history of epilepsy or seizures; metallic implants in the head or neck; concussion or head trauma within the last year; brain or spinal cord surgery; fainting spells or syncope; electric or electronic devices implanted in the body; severe skin condition which requires medical treatment and/or is painful in stimulation area; musculoskeletal injury or condition that affects the upper extremities; neurological, auditory, or vestibular condition; pregnancy or possibility of pregnancy; uncorrected visual impairment; consumption of coffee, alcohol, or other recreational drugs 10 h before the study. Sample size (*n* = 12) was determined by conducting a power analysis (*d* = 0.92, *α* = 0.05, *β* = 0.8, one sample *t*-test) on the percent improvement of root mean square error (RMSE) of a study that investigated the effects of nGVS on a unimanual tracking task in people without disabilities (Kuatsjah et al., 2019).

### Galvanic Vestibular Stimulation

**FIGURE 1:**
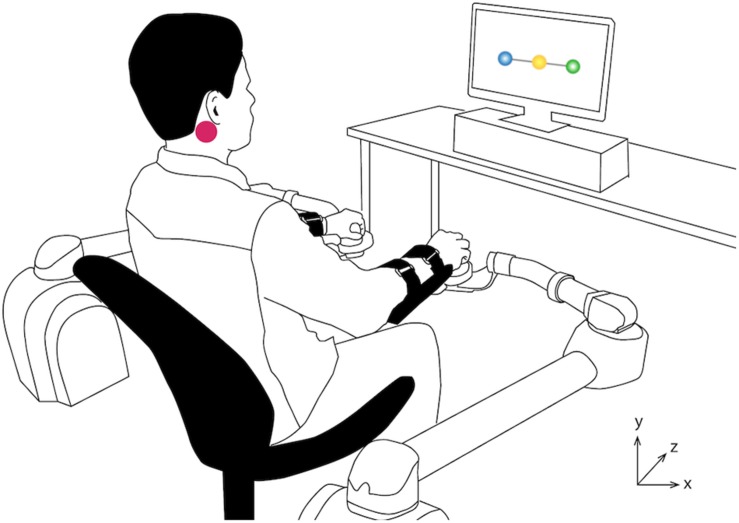
Experimental Setup and Tracking Task. A computer screen displayed the tracking task, while participants moved two robotic arms to interact with the system. Participants were required to follow the vertical movement of a target (yellow, middle circle) with their left (blue, left circle) and right (green, right circle) hands. The purple circle indicates the location of one of the electrodes.

Noisy galvanic vestibular stimulation was delivered by a constant current isolated stimulator (A395R, World Precision Instruments, FL, United States) connected to 2-inch round electrodes (ValuTrode, Axelgaard Manufacturing Co., Ltd., CA, United States). The electrodes were placed on the mastoid processes behind the participants’ ears (Figure 1). Before placing the electrodes, the skin was cleaned with alcohol prep pads (WebCol, Covidien, MA, United States).

The noisy signal was generated by a laptop computer running a custom MATLAB (MathWorks, MA, United States) script and was sent to the stimulator via the analog output of a USB data acquisition card (USB-6002, National Instruments Corporation, Austin, TX, United States). The noisy zero-mean linearly detrended stimulus had a Gaussian distribution with a 1/f-type power spectrum, which has been employed in people with and without Parkinson’s disease (Yamamoto et al., 2005; Pan et al., 2008; Kim, 2013; Kim et al., 2013; Lee et al., 2015; Kuatsjah et al., 2019). The signal was generated in the range of 0.1–10 Hz and sent to the stimulator at a rate of 60 Hz (Kim, 2013; Lee et al., 2015; Kuatsjah et al., 2019).

The stimulus was delivered below the cutaneous sensory threshold of each participant (Lee et al., 2015; Wuehr et al., 2016; Kuatsjah et al., 2019). To determine the sensory threshold, at the beginning of the session, we followed a procedure similar to the stair-case method (Wilkinson et al., 2008; Lee et al., 2015; Wuehr et al., 2016). From a starting current of 10 μA, a noisy signal was delivered for 10 s and increased in steps of 10 μA until the participant reported feeling a tingling sensation. Once the sensory threshold was set, we confirmed that the participant could not feel 90% of this value, which was the stimulation used throughout the experimental session (average peak current: 28(29) μA). In case the participant felt a tingling sensation right from the start of the thresholding procedure (10 μA), a base current of 5 μA and steps of 1 μA were employed in the stair-case method.

### Visuomotor Task

The system consisted of two BURT (Barrett Technology, MA, United States) robotic arms and a computer screen (Figure 1). The robotic arms were employed to record the position of the participants’ hands and to move the cursors in the tracking task (Figure 1). The robots did not apply any force to the participants’ hands and were free to be moved. The system was controlled by a computer running Ubuntu 16.04.3 (Canonical Group Limited, London, United Kingdom) and the tracking task was developed in Unity 5.6.2 (Unity Technologies, CA, United States) using the robots’ programing libraries. During the study, participants were seated ∼1 m away from the screen in an armless chair with their feet on top of a height-adjustable footrest and their knees at 90°.

The one-dimensional tracking task consisted of following the vertical movements of a target displayed on a computer screen (Figure 1), similar to previous studies that investigated the effects of nGVS on upper-limb fine motor performance (Lee et al., 2015; Kuatsjah et al., 2019). Participants were required to move their hands forward and backward (transverse plane, *Z* axis in Figure 1) to follow the target, while their movements were mapped to the vertical movements (*Y* axis in Figure 1) of two cursors representing each one of their hands.

**FIGURE 2:**
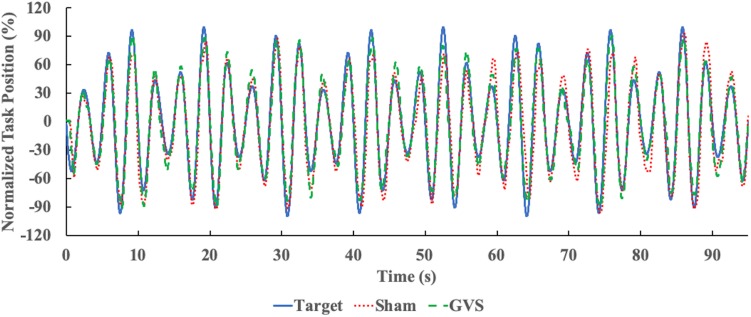
Example Target and Hand Trajectory. Target (blue, solid line) and participant’s right hand trajectory in Sham (red, dotted line) and nGVS (green, dashed line) conditions.

At the beginning of the session, the reaching task was calibrated to 80% of the distance between the participant’s dominant side hip and knee. This was done to ensure that the maximum and minimum positions of the target were achievable and consistent for all participants. During the calibration, the tracking task’s starting point was set to the midpoint between the dominant side hip and knee. Participants were asked to return to this initial position at the beginning of each trial to ensure that they always started in the same position. Once both hands were in the starting position, the computer screen displayed a message to confirm their correct placement. The target oscillated up and down following the trajectory of a sinusoidal amplitude modulated signal (Lee et al., 2015; Kuatsjah et al., 2019; Figure 2). The signal had a modulation frequency of 0.09 Hz, a carrier frequency of 0.3 Hz and a modulation index of 0.5. Four versions of the target trajectory were generated by shifting the phase and/or inverting the signal, while the frequency and modulation index remained the same (Kuatsjah et al., 2019). Trajectory 1 was the original signal, trajectory 2 was shifted 180°, trajectory 3 was inverted, and trajectory 4 was inverted and shifted 180°. The change in phase (shifted starting point) and inversion (starting oscillation going up or down) was introduced only to add some variability to the trials, while maintaining the shape of the original signal. This was intended to make it difficult for participants to predict the movement of the target in different trials.

### Experimental Procedure

**FIGURE 3:**
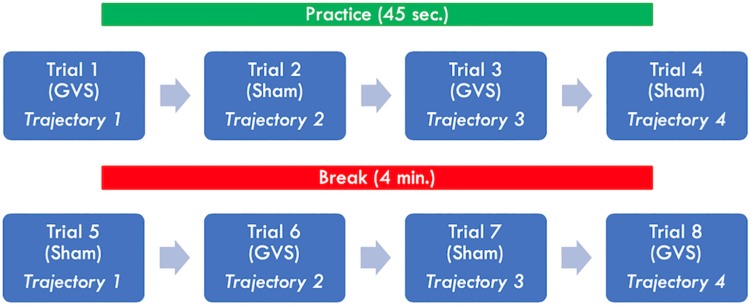
Experimental Design. Participants were allowed to practice for 45 s. Afterward, they completed 8 trials of 95 s each, with 45-s breaks between trials. A larger brake (4 min.) was provided in the middle of the study.

At the beginning of the study (Figure 3), participants were allowed to practice for 45 s with a sample trajectory (different modulation frequency and shifted from original), without receiving any stimulation. Participants were instructed to follow the target while moving both hands at the same time and in the same direction (forward/backward). After the practice trial, participants completed 8 trials of 95 s each, with 45-s breaks between trials. A larger break (4 min) was taken in the middle of the study to provide participants with additional rest time. During the breaks, it was confirmed that the electrodes were in place and properly attached. In four of the trials, participants received nGVS stimulation, and in the remaining four, they received a sham stimulation. Trials were arranged such that a trajectory was not immediately repeated, while ensuring that all trajectories were completed in the stimulation and sham conditions (Figure 3). In addition, the order of stimulation conditions was alternated (Figure 3). Participants were blind to the order in which they received stimulation and the electrodes were attached at all times.

### Data Analysis

Position data from the target and cursors were used for the analysis. Each tracking trial duration was 95 s, sampled at ∼60 Hz, and the first 5 s of data were excluded to reduce the effect of initial adaptation of the participant to the task (Kuatsjah et al., 2019).

The primary outcome measure was the percent improvement of the RMSE between the target’s and cursors’ trajectories, which provided us with a spatial measure of the error in the tracking task. One of the secondary outcome measures was the percent improvement of the lag between the cursors and target, which allowed us to explore the temporal error that participants exhibited. This was calculated using the lag parameter of MATLAB’s cross-correlation function (xcorr), which defines lag as the shift (number of samples) between two signals that results in the maximum cross-correlation (MathWorks, 2019). This method has been employed to measure lag in tracking tasks (Foulkes and Miall, 2000; Heitger et al., 2004). The other secondary outcome measure was the percent improvement of the RMSE between the cursors’ trajectories, which provided us with a spatial measure of the bimanual coordination error in the tracking task. Percent improvement was calculated as the ratio of the difference between the average of the Sham trials (2, 4, 5, 7) and the average of nGVS trials (1, 3, 6, 8) to the average of the Sham trials, of each participant, with a positive percentage indicating an improvement of performance in the nGVS condition.

Given that an increased percent improvement in RMSE during the first 30 s of nGVS in a unimanual tracking task has been observed (Kuatsjah et al., 2019), we performed an exploratory analysis to investigate how the primary outcome measure changed throughout the duration of the trials by analyzing the data in three 30-s subsections.

A post-test questionnaire was administered at the end of the study session to evaluate the experience of participants with the tracking task, nGVS, and the robotic system.

### Statistical Analysis

To investigate if nGVS had an effect on RMSE between the cursors and the target (primary outcome measure), and between cursors (secondary outcome measure), a one-sample *t*-test was conducted on the percent improvements against a mean value of 0. To evaluate if the effect of nGVS on one hand was more pronounced when compared with the other one, a dependent *t*-test between the percent gains of the left and right hands was employed. Lag was analyzed in a similar manner. When violations to models’ assumptions were observed, the less restrictive Sign Test was employed. For the exploratory analysis of the 3 blocks of 30-s primary outcome RMSE data, a two-way repeated measures ANOVA was conducted with factors of time block and hand. All statistical tests and models’ assumptions were processed in SPSS Statistics v24.0 (IBM Corp., Armonk, NY, United States).

## Results

**FIGURE 4:**
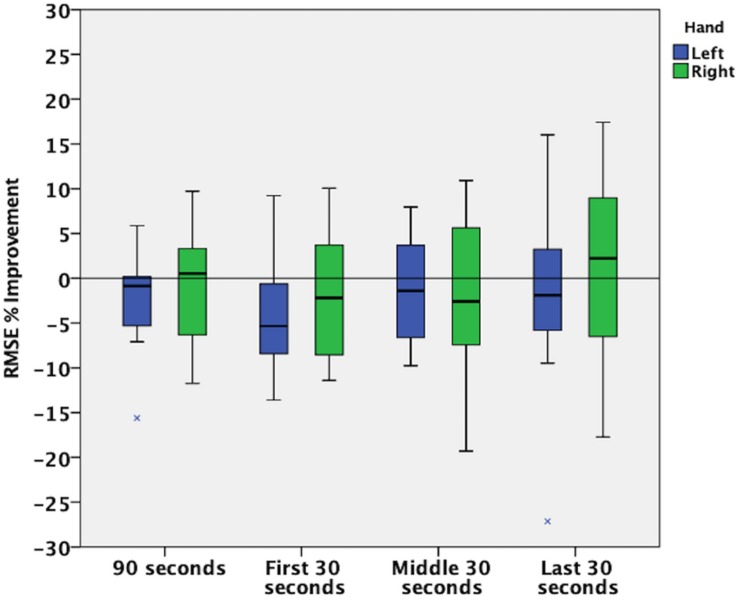
Percent Improvement in Root Mean Square Error (RMSE).

During the whole duration of the trials, the error between the hands’ position and the target was not affected by receiving nGVS (left −2.6(5.5)%, *t*(11) = −1.648, *p* = 0.128; right −0.9(6.2)%, *t*(11) = −0.482, *p* = 0.639), as shown in Figure 4 and Table 1. In addition, when comparing if one of the hands’ RMSE changed more than the other (left-right), we did not find a statistically significant difference (mean difference: −1.7(3.3)%, *t*(11) = −1.805, *p* = 0.098).

**TABLE 1:** Root mean square error percent improvement.

	**RMSE (% improvement)**							
	
	**90 s**		**First 30 s**		**Middle 30 s**		**Last 30 s**	
				
	**L**	**R**	**L**	**R**	**L**	**R**	**L**	**R**
P1	–0.8	1.6	–5.9	–0.6	–6.3	–3.9	8.6	8.2
P2	–7.1	–5.5	–13.6	–10.4	–7.6	–19.3	0.2	10.8
P3	–5.5	3.0	–9.0	5.9	–0.7	–2.2	–6.7	3.3
P4	–0.8	–0.6	–7.9	–7.9	7.9	9.2	–3.9	–5.3
P5	–3.9	–8.1	0.1	–1.6	–5.5	–6.8	–4.9	–14.0
P6	–1.0	–1.1	–1.4	–2.9	–2.1	–1.3	0.7	1.2
P7	0.9	4.0	9.2	10.1	–7.0	–8.1	1.8	9.7
P8	–15.6	–11.7	–11.0	–7.6	–9.8	–10.4	–27.1	–17.7
P9	2.4	1.9	–4.8	–9.2	5.7	7.2	4.7	4.5
P10	–5.1	–7.2	–7.7	–11.4	1.3	–2.9	–9.5	–7.8
P11	–0.5	3.7	3.0	9.0	1.7	4.1	–4.7	–0.9
P12	5.9	9.7	–3.4	1.6	6.0	10.9	16.0	17.4

Average	–2.6	–0.9	–4.4	–2.1	–1.4	–2.0	–2.1	0.8
SD	5.5	6.2	6.4	7.5	6.0	8.8	10.6	10.5
Median	–0.9	0.5	–5.3	–2.2	–1.4	–2.6	–1.9	2.2
Q1	–5.4	–6.8	–8.7	–8.9	–6.8	–7.8	–6.2	–7.1
Q3	0.5	3.5	–0.3	4.8	4.7	6.4	3.9	9.4
IQR	5.9	10.3	8.4	13.7	11.5	14.2	10.2	16.5

*IQR, interquartile range; L, left; Q1, 1st quartile; Q3, 3rd quartile; R, right; RMSE, root mean square error.*

The lag between the hands and the target (Table 2) did not change when receiving nGVS [left median (Q1 and Q3) = −10.4 (−18.1, 0.0)%, *sign test p* = 0.109; right −4.5(−15.1, 7.0)%, *sign test p* = 0.753], nor did we find that one hand was affected more than the other [left-right, median difference: −0.8 (−7.0, 10.8), *sign test p* = 1.0].

**TABLE 2:** Lag percent improvement.

	**Lag (% improvement)**	
	
	**90 s**	
	
	**L**	**R**
P1	–6.1	–9.1
P2	–11.8	–14.3
P3	100.0	16.7
P4	0.0	7.7
P5	–16.7	–14.3
P6	–12.5	0.0
P7	–70.6	–86.7
P8	–22.6	–23.3
P9	–9.1	0.0
P10	–18.5	–15.4
P11	30.0	16.7
P12	0.0	4.8

Average	–3.1	–9.8
SD	39.7	27.4
Median	–10.4	–4.5
Q1	–18.1	–15.1
Q3	0.0	7.0
IQR	18.1	22.1

*IQR, interquartile range; L, Left; Q1, 1st quartile; Q3, 3rd quartile; R, right.*

The bimanual coordination of participants was not affected by the application of nGVS [−1.5(9.6)%, *t*(11) = −0.555, *p* = 0.590], as shown in Table 3.

**TABLE 3:** Right vs. left cursor root mean square error percent improvement.

	**RMSE (% improvement)**
	
	**90 s**
	
	**R – L Cursors**
P1	18.3
P2	–1.8
P3	2.0
P4	–10.4
P5	–13.4
P6	–1.1
P7	4.3
P8	–1.0
P9	6.3
P10	–11.9
P11	4.1
P12	–13.6

Average	–1.5
SD	9.6
Median	–1.1
Q1	–11.5
Q3	4.3
IQR	15.8

*IQR, interquartile range; L, left; Q1, 1st quartile; Q3, 3rd quartile; R, right; RMSE, root mean square error.*

When exploring if RMSE was more affected at the beginning, middle, or end of the trial, we did not find a statistically significant result for the main effects of time [*F*(2,22) = 0.402, *p* = 0.674] and hand [*F*(1,11) = 2.645, *p* = 0.132], nor for the interaction between them [*F*(2,22) = 1.717, *p* = 0.203]. Results are shown in Table 1 and Figure 4.

**FIGURE 5:**
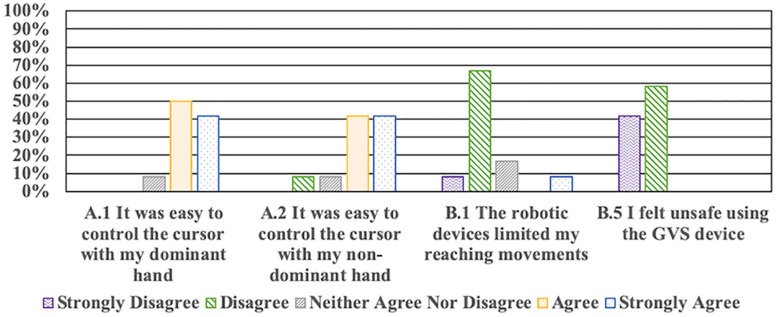
Post-test questionnaire selected results. Remaining questions are presented in the Supplementary Material.

Post-test questionnaire results are presented as Supplementary Material, and a selection of questions are shown in Figure 5. When asked if the virtual cursors were easy to control with both their dominant and non-dominant hands, the majority of participants agreed or had a neutral response (100% dominant, 91.7% non-dominant). When investigating if the robots limited the reaching movements of participants, the majority disagreed or had a neutral response (91.7%). All participants (100%) reported not feeling unsafe when using the GVS device. To investigate a possible negative effect of stimulating with nGVS, we asked participants if they felt any discomfort during the session, with 100% reporting no discomfort. After every trial, participants were asked if they felt any stimulation. For sham trials, 75% participants correctly reported not feeling stimulation in all trials (4/4), and 25% not feeling in 3/4 trials. For nGVS trials, 41.6% reported not feeling any stimulation in all trials (4/4), 8.3% felt stimulation in 1/4 trials, 41.6% in 2/4 trials, and 8.3% in 3/4 trials. No participant was able to correctly identify all nGVS and sham trials.

## Discussion

This study was conducted to investigate the effects of nGVS in gross upper-limb motor performance during a bimanual tracking task. Previous work in people with and without Parkinson’s disease (Kim, 2013; Lee et al., 2015; Kuatsjah et al., 2019), reported improvements in fine upper-limb unimanual motor performance in similar tracking tasks. Stochastic resonance has been proposed as one of the possible mechanisms that lead to improved performance, not only in these fine upper-limb motor studies, but also in walking (Wuehr et al., 2016; Iwasaki et al., 2018) and standing balance investigations (Iwasaki et al., 2014; Inukai et al., 2018). In this study, we did not find any statistically significant positive nor negative effects (spatial or temporal) of applying nGVS at 90% of participants’ sensory threshold. Moreover, we did not find that one arm was more affected by nGVS than the other. However, in the scope of this study, we only tested one sensory threshold percentage and employed specific noise characteristics that were based on previous upper-limb fine motor studies (Lee et al., 2015; Kuatsjah et al., 2019). In the case of gross arm movements comprised of larger muscle recruitment and increased ranges of motion, the optimal threshold and noise characteristics of the stimulation signal might be different. Furthermore, alternative nGVS parameters might be needed for tasks that involve simultaneous use of both upper limbs, as the hemispheric inhibition/excitation patterns (e.g., reduced intracortical inhibition in both hemispheres) and brain structures’ activation (e.g., corpus callosum involved in bilateral spatial coupling) involved in bimanual tasks can be different than those for unimanual tasks (Cauraugh and Summers, 2005; McCombe Waller and Whitall, 2008). At this stage we can only speculate on the reasons behind the different nGVS effects observed in this behavioral study. Future studies should investigate the effects of nGVS when participants perform unimanual gross movements, in addition to bimanual fine motor movements, to be able to shed more light on how noisy stimulation might affect performance when one or two hands are recruited, and when small and large arm/hand movements are required. There is also evidence to suggest that an optimal level of noisy current intensity can lead to a positive effect, but a further increase can lead to diminished performance (Iwasaki et al., 2014). As such, different stimulation levels and their relationship to upper-limb gross motor performance should be investigated in future studies.

In the literature, studies in individuals without disabilities have also shown no effect of GVS on unimanual tracking (Dilda et al., 2012) or detrimental effects on arm position sense (Schmidt et al., 2013a), using pseudo-random and direct current stimulation, respectively. These studies, in combination with our results, suggest that the stimulation parameters and nature of the task could play a substantial role in the observed positive or negative effects of GVS. This calls for further exploration of the effects of GVS on different motor tasks, as a “one-size-fits-all” stimulation for upper-limb motor tasks might be non-existent.

A previous study found larger improvement effects of nGVS during the first 30 s of application (Kuatsjah et al., 2019). Our exploratory study found a similar, but opposite, trend with larger median detriments in the first 30 s compared to the last 30 s. This might also support the idea that stimulation parameters employed in fine motor unimanual paradigms might be non-optimal or have an opposite effect when performing bimanual gross motor tasks. The observation that nGVS appears to have a larger effect on the first seconds of exposure, might suggest that its effects are reduced when habituation to the task, decreased attention, or fatigue occurs (Kuatsjah et al., 2019). However, this hypothesis should be first confirmed with additional studies exploring different upper-limb reaching paradigms and longer task durations.

As previously mentioned, stochastic resonance has been hypothesized as a possible mechanism for motor improvement during the application of nGVS (Kim, 2013). However, in order to gain insight into the exact neural mechanisms and areas of the brain that are affected by nGVS during a visuomotor task, further studies should be conducted in which brain activity is recorded while participants receive vestibular stimulation and complete a tracking task. fMRI studies (Lobel et al., 1998; Bense et al., 2001; Stephan et al., 2005; Cai et al., 2018) with alternating, direct, and noisy current GVS applied to participants, in a supine position with eyes closed, have shown activation of diverse subcortical and cortical structures, such as areas of the: cerebellum; posterolateral and paramedian thalamus; supplementary motor area; pons; putamen; insular gyri; middle, inferior and superior temporal gyri; inferior and middle frontal gyri; supramarginal gyrus; lateral and central sulcus; cingular gyrus; and precentral gyrus and sulcus, amongst others. In addition, in an electroencephalography study, modulation of brain rhythms (theta, alpha, beta, and gamma frequency bands) was observed when people without disabilities received nGVS in an eyes-open resting state, which has been proposed as a possible mechanism for motor and cognitive effects resulting from vestibular stimulation (Kim et al., 2013). In a behavioral visuomotor study in individuals with Parkinson’s disease (Lee et al., 2015), the authors provided several hypotheses regarding the mechanisms of nGVS, such as enhancement of cingulate activity, and as a result, regulation of fronto-midline theta activity; modulation of cortico-basal ganglia rhythms; and stochastic resonance modulating the transmission and detection of stimuli in the sensorimotor system. Together, these studies provide support to the idea that through vestibular stimulation, we can gain access to and perhaps regulate the activity of subcortical and cortical areas of the brain that could be involved in the perception, planning, and execution of movements. This is an appealing concept given that nGVS is a relatively safe and non-invasive technique, with effects that can be observed even at low levels of current (Kim, 2013; Inukai et al., 2018; Temple et al., 2018). However, there is still a need for more mechanistic studies that explore the relation between the influence of nGVS on upper-limb motor performance and concurrent brain activation patterns, before a conclusion about its exact effects can be reached.

Based on post-test questionnaire results, the robotic devices did not limit the required reaching movements, did not make participants feel unsafe, nor did they were difficult to control to interact with the task. As such, it would appear that the lack of effect of nGVS on motor performance was not the result of the testing setup. In addition, participants did not feel unsafe with the GVS device, nor did they report any discomfort during the session due to the stimulation. This in agreement with previous studies that have reported no or only mild adverse effects when employing GVS (Wilkinson et al., 2009; Utz et al., 2011; Schmidt et al., 2013a). As such, the technology could be employed as a safe tool for neuromodulation.

### Limitations

Given that older adults can exhibit age-related motor and brain deficits (Seidler et al., 2010), the question of how this population’s gross motor performance is affected by nGVS should be investigated in future studies, as stimulation parameters might differ from the ones employed in this work. Additionally, this study only investigated the short-term effects of nGVS. However, exploring its long-term effects is important (Kim, 2013), particularly before this technology can be widely promoted to potentially improve motor performance.

There were some differences between the methodology followed by Kuatsjah (Kuatsjah et al., 2019) and our study. Kuatsjah employed a custom measure of lag, while we decided to use a cross-correlation function, which is a standard measure that has been employed in previous studies (Foulkes and Miall, 2000; Heitger et al., 2004). We do not believe that this contributed to the lack of observed effect of the temporal error, as both measures provided an indication of the time shift of the tracking signals. However, future studies might benefit from employing additional measures of temporal error. Another difference was that Kuatsjah employed DC signals to test for the sensory threshold of participants. In our study we chose to use a nGVS signal, as this was the same type of signal that participants received during the experimental trials, which could have allowed for a better blinding of participants to the sham and stimulation trials. Given that Kuatsjah employed DC signals for measuring participants’ threshold values, this could have resulted in participants receiving current values above their sensory threshold during the experimental trials, as the noise values would have peaks above the DC threshold values. This difference in the thresholding stimulation signal might have led to the lower current values recorded in our study.

In terms of suggested improvements to the thresholding procedure, researchers might want to confirm that participants are feeling a tingling sensation for the whole duration of the nGVS current steps. In this study, we asked participants to indicate to us as soon as they felt a tingling sensation, which could have led to setting lower thresholding currents. In addition, future studies might benefit from having the thresholding procedure take place at the same time as participants are completing a task that mimics the movements that they will be performing during the experimental trials. With the thresholding method we employed, it could be possible that participants were more sensitive to feeling a tingling sensation while they were resting, as their full attention is employed at identifying any cutaneous sensation. On the other hand, if participants are asked to perform a task, while receiving the current steps, they might not notice any sensation, which could enable researchers to employ higher current values.

The tracking task included two cursors of different colors that represented the participants’ hands. We chose to represent the cursors with different colors to allow participants to easily differentiate between the two. However, this could have induced a potential attention bias toward one of the cursors. Future studies could investigate if employing the same color for both cursors might lead to different results.

The sample of this study only included participants without neurological disorders. As such, the participants might have been already too proficient at performing the bimanual reaching task, which could have contributed to the lack of performance improvement. If participants without neurological disorders are to be employed in future studies, perhaps a bimanual tracking task with a higher level of difficulty should be employed to investigate possible stimulation effects. In addition, the effects of nGVS on gross motor movements might be different in populations in which areas of the brain related to the planning and execution of motor commands is damaged or deteriorated (e.g., stroke and Parkinson’s), as it has been speculated that the effectiveness of GVS might be related to disease severity (Cai et al., 2018).

## Conclusion

The results from this study suggest that nGVS applied to people without disabilities do not result in significant changes to bimanual gross motor performance. However, as this was the first study to investigate such effects, the stimulation parameters were based on previous unimanual fine motor studies that found improvements when applying nGVS. Future studies should investigate the optimal stimulation parameters for improving upper-limb gross motor performance. Overall, participants felt safe using the robotic devices and receiving the noisy electrical stimulation. As such, a similar setup could be employed for subsequent studies investigating the relation between upper-limb performance and nGVS.

## Data Availability Statement

The datasets used and/or analyzed during the current study are available from the corresponding author on reasonable request.

## Ethics Statement

The studies involving human participants were reviewed and approved by the Research Ethics Board, Simon Fraser University. The participants provided their written informed consent to participate in this study.

## Author Contributions

BV and CM conceived and designed the experiments, and reviewed and edited the manuscript. BV implemented the experimental setup, collected the data, analyzed the data, and wrote the manuscript.

## Conflict of Interest

The authors declare that the research was conducted in the absence of any commercial or financial relationships that could be construed as a potential conflict of interest. The reviewer MM declared a past co-authorship with one of the authors, CM, to the handling Editor.

## Abbreviations

fMRI: functional magnetic resonance imaging
GVS: galvanic vestibular stimulation
nGVS: noisy galvanic vestibular stimulation
RMSE: root mean square error.
