# Supplementary material for: Effects of Noisy Galvanic Vestibular Stimulation During a Bimanual Tracking Robotic Task
Source: Front Neurosci. 2019 Oct 25;13:1132. doi: 10.3389/fnins.2019.01132 (PMC6843009; doi:10.3389/fnins.2019.01132)

POST-TEST QUESTIONNAIRE RESULTS

POST-TEST QUESTIONNAIRE PART A (REACHING TASK)

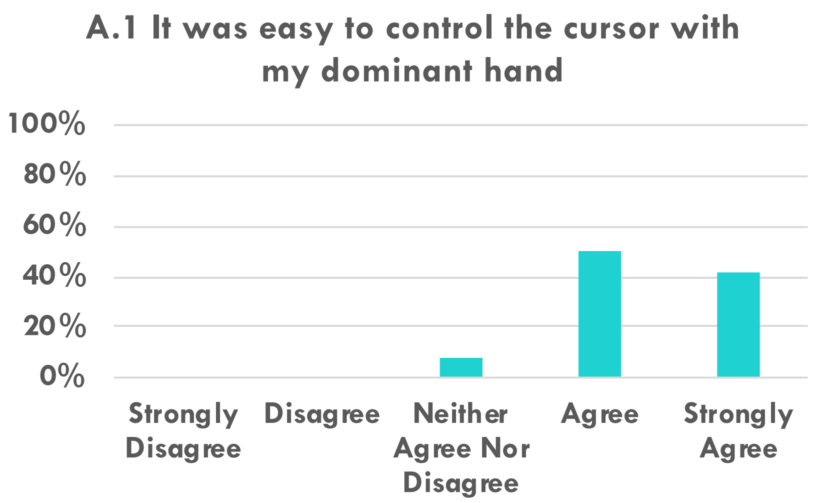

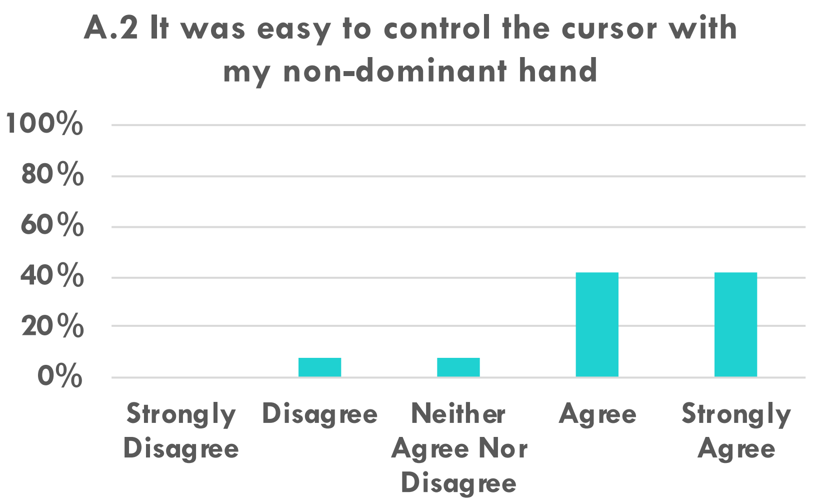

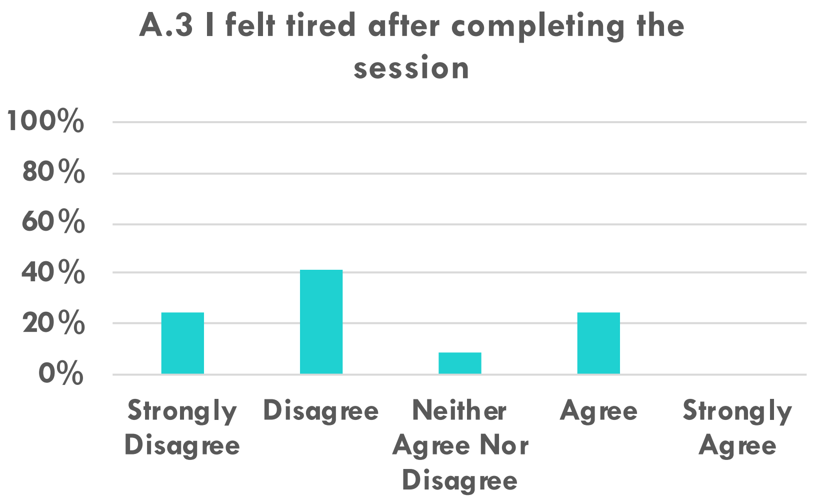

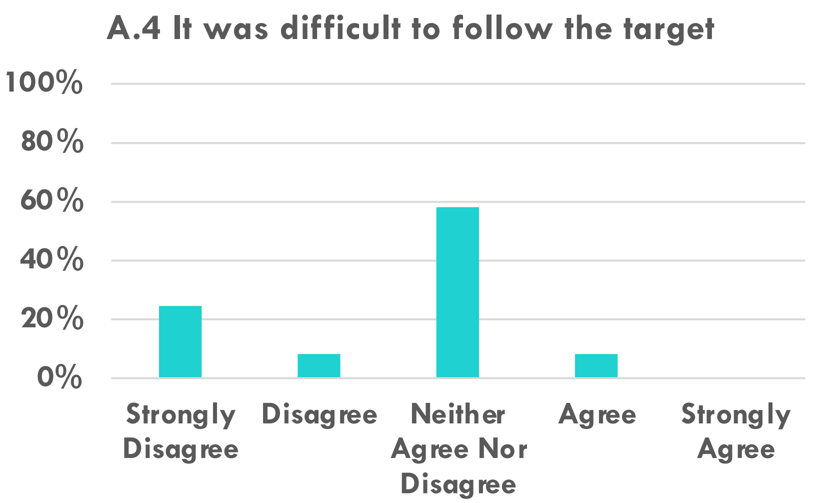

POST-TEST QUESTIONNAIRE PART B (SYSTEM DESIGN)

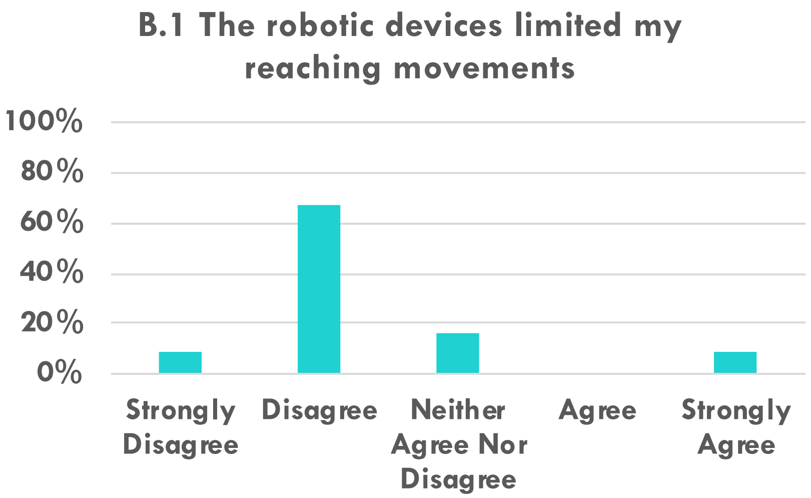

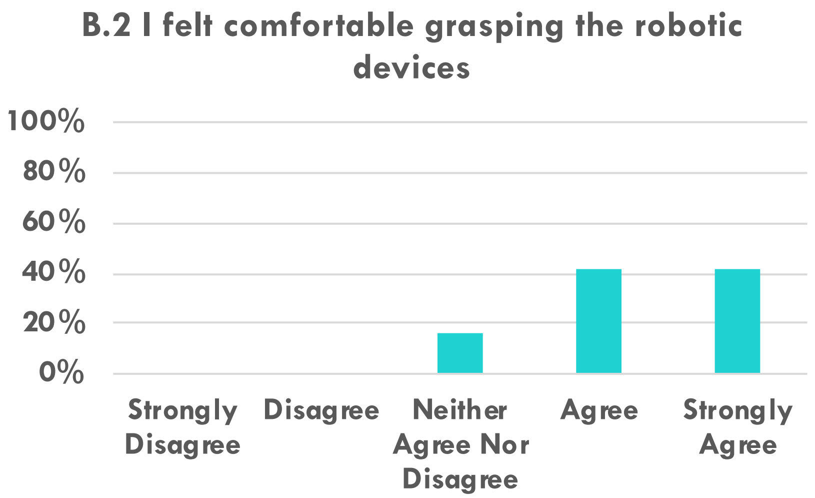

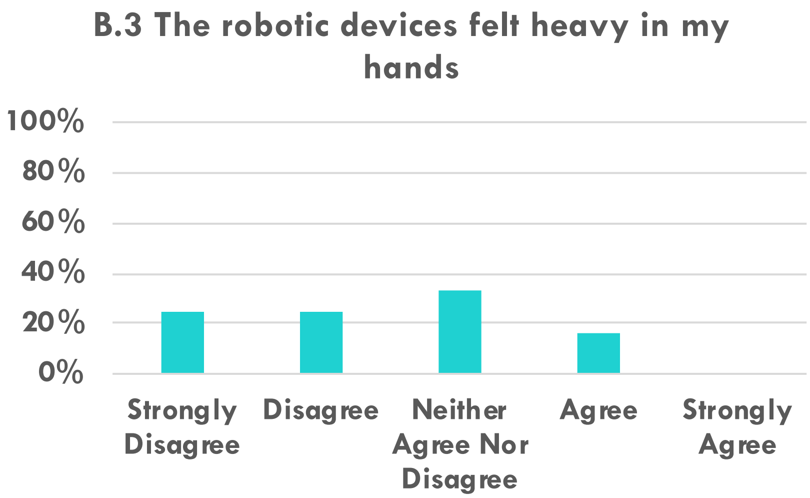

POST-TEST QUESTIONNAIRE PART B (SYSTEM DESIGN)

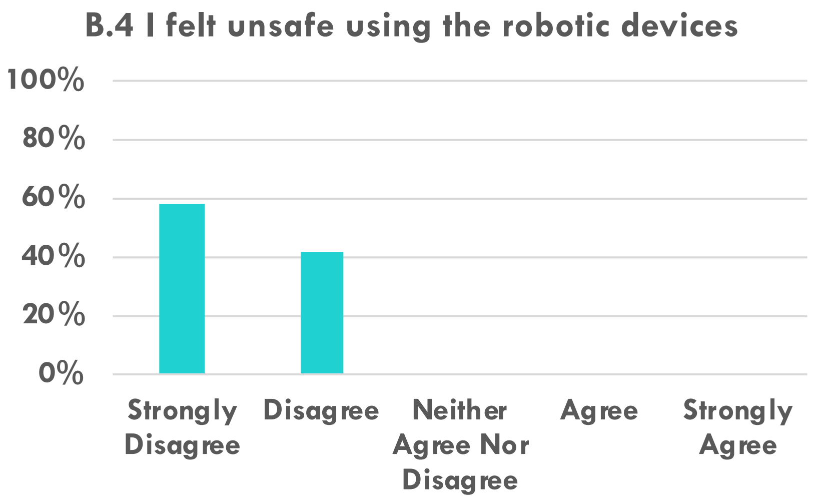

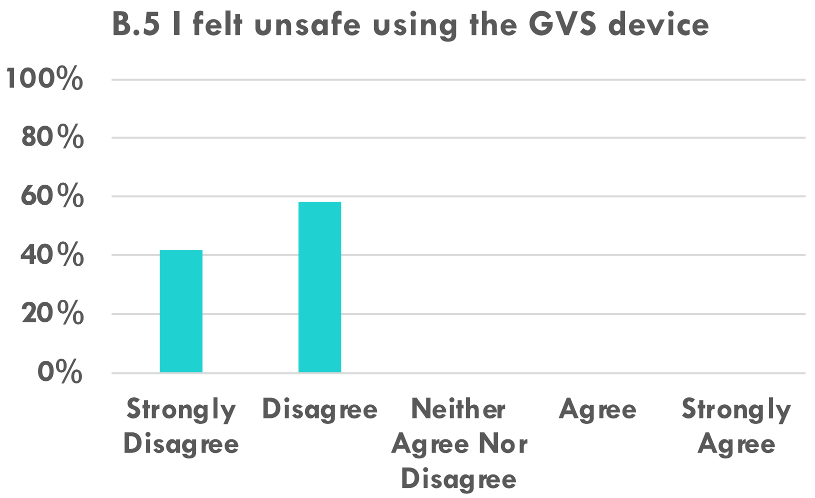

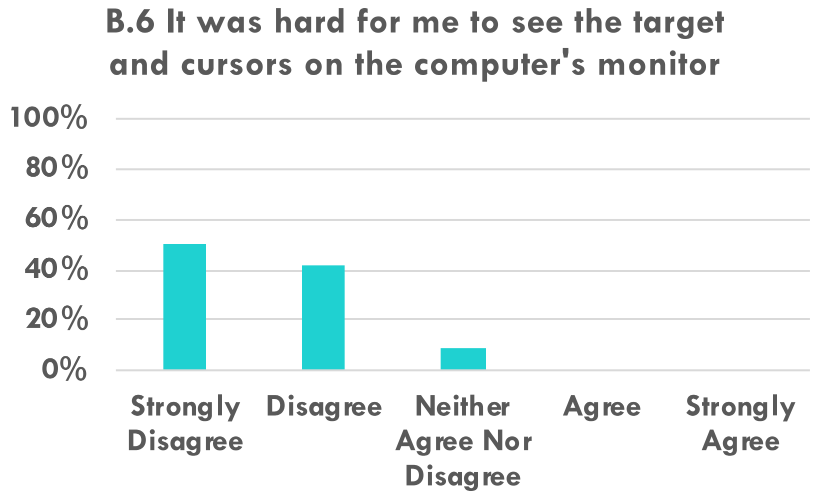

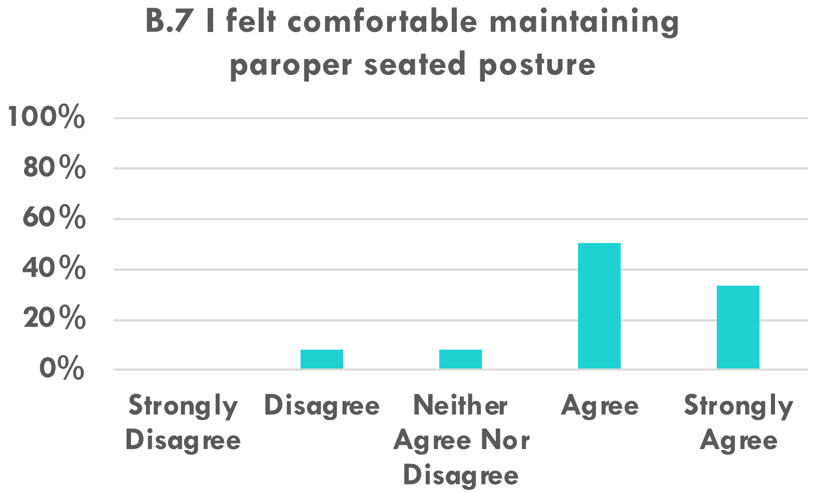

POST-TEST QUESTIONNAIRE PART C (GVS VS SHAM)

Did you feel any galvanic vestibular stimulation (after every trial)?											
**Trial Number**	**1**	**2**	**3**	**4**	**5**	**6**	**7**	**8**			
**Correct Answer (Yes/GVS=1, No/Sham=2)**	**1**	**2**	**1**	**2**	**2**	**1**	**2**	**1**	**GVS Correct %**	**Sham Correct %**	**Total Correct %**
**P01**	2	2	2	2	2	2	2	2	0.0%	100.0%	50.0%
**P02**	2	2	1	2	2	2	2	1	50.0%	100.0%	75.0%
**P03**	2	1	2	2	2	1	2	1	50.0%	75.0%	62.5%
**P04**	2	2	2	2	2	2	2	2	0.0%	100.0%	50.0%
**P05**	2	2	2	2	2	2	2	2	0.0%	100.0%	50.0%
**P06**	2	2	1	2	2	2	1	1	50.0%	75.0%	62.5%
**P07**	2	2	2	2	2	2	2	2	0.0%	100.0%	50.0%
**P08**	2	2	1	2	1	1	2	2	50.0%	75.0%	62.5%
**P09**	2	2	2	2	2	2	2	2	0.0%	100.0%	50.0%
**P10**	2	2	1	2	2	2	2	1	50.0%	100.0%	75.0%
**P11**	1	2	1	2	2	2	2	1	75.0%	100.0%	87.5%
**P12**	1	2	2	2	2	2	2	2	25.0%	100.0%	62.5%

POST-TEST QUESTIONNAIRE PART D (DISCOMFORT)

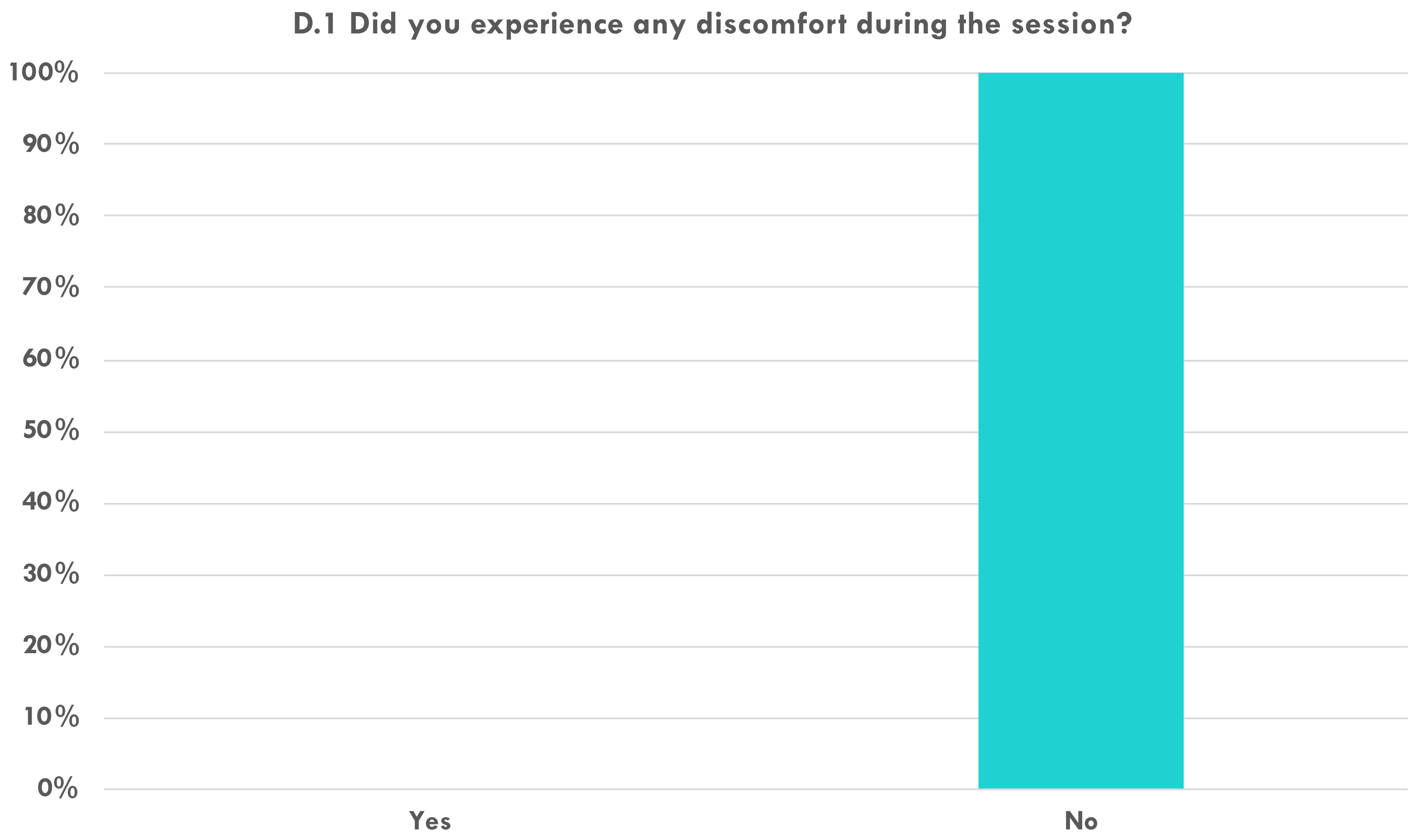

Supplement: Supplementary file 1 [file Data_Sheet_1.PDF]
